# Supplementary material for: Epidemiology of extended-spectrum beta-lactamase-producing Escherichia coli at the human-animal-environment interface in a farming community of central Uganda
Source: PLOS Glob Public Health. 2023 Jun 13;3(6):e0001344. doi: 10.1371/journal.pgph.0001344 (PMC10270331; doi:10.1371/journal.pgph.0001344)
Supplement: S1 File — (PDF) [file pgph.0001344.s001.pdf]

Uganda National Council for Science and Technology
(Established by Act of Parliament of the Republic of Uganda)

Our Ref: HS1919ES **20 January 2022**

JAMES MULEME
Makerere University School of Public Health
Kampala

Re: Research Approval: **Reservoirs, transmission and antibiotic resistance profiles of Extended Spectrum Beta Lactamase-producing Escherichia Coli at the human-animal-environment interface among farming communities in\r\nWakiso district, Uganda\r\n**

I am pleased to inform you that on **20/01/2022**, the Uganda National Council for Science and Technology (UNCST) approved the above referenced research project. The Approval of the research project is for the period of **20/01/2022** to **20/01/2026**.

Your research registration number with the UNCST is **HS1919ES**. Please, cite this number in all your future correspondences with UNCST in respect of the above research project. As the Principal Investigator of the research project, you are responsible for fulfilling the following requirements of approval:

1. Keeping all co-investigators informed of the status of the research.
2. Submitting all changes, amendments, and addenda to the research protocol or the consent form (where applicable) to the designated Research Ethics Committee (REC) or Lead Agency for re-review and approval **prior** to the activation of the changes. UNCST must be notified of the approved changes within five working days.
3. For clinical trials, all serious adverse events must be reported promptly to the designated local REC for review with copies to the National Drug Authority and a notification to the UNCST.
4. Unanticipated problems involving risks to research participants or other must be reported promptly to the UNCST. New information that becomes available which could change the risk/benefit ratio must be submitted promptly for UNCST notification after review by the REC.
5. Only approved study procedures are to be implemented. The UNCST may conduct impromptu audits of all study records.
6. An annual progress report and approval letter of continuation from the REC must be submitted electronically to UNCST. Failure to do so may result in termination of the research project.

Please note that this approval includes all study related tools submitted as part of the application as shown below:

No.	Document Title	Language	Version Number	Version Date
1	Study tools	English and Luganda	1	05 January 2022
2	Project Proposal	English	FINAL	
3	Approval Letter	English		
4	Administrative Clearance	English		
4	Letter from Lab 1	English	1	19 January 2022
5	Letter from Lab 2	English	1	19 January 2022
6	Letter from study district	English	1	19 January 2022
7	Approved_Biorisk management plan	English	1	19 January 2022
8	Covid19-Risk mitigation plan	English	1	19 January 2022
9	Laboratory request form	English	1	19 January 2022
10	Clean document word version	English	1	19 January 2022
11	Muleme James Signed CV	English	1	19 January 2022
12	Sanitary Inspection tool	English	1	19 January 2022
13	Questionnaire_Eng	English	1	19 January 2022
14	Questionnaire_Lug	Luganda	1	19 January 2022
15	KII guide for professionals	English	1	19 January 2022
16	KII guide for regulators	English	1	19 January 2022
17	Consent form_Eng	English	1	19 January 2022
18	Consent form_Lug	Luganda	1	19 January 2022
19	Assent form_Eng	English	1	19 January 2022
20	Assent form_Lug	Luganda	1	19 January 2022
21	Focus Group discussion guide_Eng	English	1	19 January 2022
22	Focus Group discussion guide_Lug	Luganda	1	19 January 2022
23	Consent form_FGD_Eng	English	1	20 January 2022
24	Consent form_FGD_Lug	Luganda	1	20 January 2022
25	Consent form_KII_Eng	English	1	20 January 2022
26	Consent form_KII_Lug	Luganda	1	20 January 2022

Yours sincerely,

Hellen Opolot
For: Executive Secretary
UGANDA NATIONAL COUNCIL FOR SCIENCE AND TECHNOLOGY

MAKERERE UNIVERSITY
P.O. Box 7072 Kampala Uganda
Website: http://www.musph.ac.ug
Tel: 256-41-532207/543872/543437
Fax: 256-41-531807

COLLEGE OF HEALTH SCIENCES

SCHOOL OF PUBLIC HEALTH

HIGHER DEGREES, RESEARCH AND ETHICS COMMITTEE

14/12/2021

To: JAMES MULEME

Makerere University School of Public Health
0787364697

Type: Initial Review

Re: SPH-2021-167: Reservoirs, transmission and antibiotic resistance profiles of Extended Spectrum Beta Lactamase-producing Escherichia Coli at the human-animal-environment interface among farming communities in Wakiso district, Uganda, CV for proposed supervisor, 2021-11-20

I am pleased to inform you that at the **195** convened meeting on **14/12/2021**, the MAK School of Public Health REC (SPHREC), committee meeting, etc voted to approve the above referenced application.
Approval of the research is for the period of **14/12/2021** to **14/12/2022.**

As Principal Investigator of the research, you are responsible for fulfilling the following requirements of approval:

1. All co-investigators must be kept informed of the status of the research.
2. Changes, amendments, and addenda to the protocol or the consent form must be submitted to the REC for re-review and approval **prior** to the activation of the changes.
3. Reports of unanticipated problems involving risks to participants or any new information which could change the risk benefit: ratio must be submitted to the REC.
4. Only approved consent forms are to be used in the enrollment of participants. All consent forms signed by participants and/or witnesses should be retained on file. The REC may conduct audits of all study records, and consent documentation may be part of such audits.
5. Continuing review application must be submitted to the REC **eight weeks** prior to the expiration date of **14/12/2022** in order to continue the study beyond the approved period. Failure to submit a continuing review application in a timely fashion may result in suspension or termination of the study.
6. The REC application number assigned to the research should be cited in any correspondence with the REC of record.
7. You are required to register the research protocol with the Uganda National Council for Science and Technology (UNCST) for final clearance to undertake the study in Uganda.

The following is the list of all documents approved in this application by MAK School of Public Health REC (SPHREC):

No.	Document Title	Language	Version Number	Version Date
1	Protocol	English	CV for proposed supervisor	2021-11-20
2	Protocol	English	Letter to request change of one supervisor	2021-11-20
3	Informed Consent forms	English and Luganda	1	2021-10-09
4	Data collection tools	English and Luganda	1	2021-10-09
5	Protocol	English	1	2021-10-09

Yours Sincerely

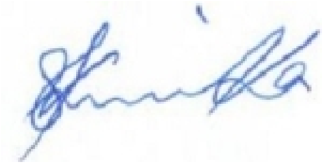

Suzanne Kiwanuka
For: MAK School of Public Health REC (SPHREC)

WAKISO DISTRICT LOCAL GOVERNMENT

Office of the Chief Administrative Officer
P.O. Box 7218,Kampala Uganda ,Tel: +256 392 723334
Email :wakisodlc@yahoo.co.uk / Website:www.wakiso.go.ug

THE REPUBLIC OF UGANDA

TOGETHER WE WIN

REF CRD 220/1

Date: 13th January 2022

Mr Muleme James (PHD Candidate)
Makerere University

PERMISSION TO CARRY OUT RESEARCH

Reference is made to your letter dated 10th January 2022 requesting for permission to carry out research in Wakiso District on the topic "**Reservoir and transmission dynamics of extended spectrum Beta Lactamase producing Escherichia coli at the human-animal environment interface in Wakiso district, Uganda"**

Permission is hereby granted to you to carry out your research in Wakiso District. You are advised to ensure that the information/data collected is used for academic purposes only. You should also ensure that you submit a copy of your research report to this office.

By copy of this letter all Heads of Departments are requested to accord you the necessary assistance.

For CHIEF ADMINISTRATIVE OFFICER
13 JAN 2022

Byamukama Alfred
FOR: CHIEF ADMINISTRATIVE OFFICER

DISTRICT HEALTH OFFICER
13 JAN 2022
RECEIVED

Copy to: The Research Supervisor – Makerere University
District Health Officer – Wakiso District
The District Production Officer– Wakiso District

Received by Sec production for DPO
13/1/2022
