# Supplementary material for: Epidemiology of extended-spectrum beta-lactamase-producing Escherichia coli at the human-animal-environment interface in a farming community of central Uganda
Source: PLOS Glob Public Health. 2023 Jun 13;3(6):e0001344. doi: 10.1371/journal.pgph.0001344 (PMC10270331; doi:10.1371/journal.pgph.0001344)
Supplement: S2 File — (PDF) [file pgph.0001344.s002.pdf]

MAKERERE UNIVERSITY

DIRECTORATE OF RESEARCH AND GRADUATE TRAINING

P. O. Box 7062, Kampala Uganda
Tel: +256 414 530983
Fax: +256 414 533809

E-mail: drgt@rgt.mak.ac.ug
Website: www.mak.ac.ug

REG.NO **2020/HD07/24864U**

Date **21st January, 2022**

NAME **MULEME James**

Dear Sir/Madam,

RE: <u>FULL ADMISSION TO Ph.D. DEGREE STUDIES</u>

Following successful vetting of your Ph.D. research proposal, I am pleased to inform you that you have been offered **Full Admission** status on a programme of study leading to the award of the Degree **Doctor of Philosophy** of Makerere University.

Your registration is by research and Thesis effective from **19th January, 2022** and expires on **20th January, 2025**.

Your approved research topic is:

"RESERVOIRS, TRANSMISSION AND ANTIBIOTIC RESISTANCE PROFILES OF EXTENDED SPECTRUM BETA LACTAMASE-PRODUCING ESCHERICHIA COLI AT THE HUMAN-ANIMAL ENVIRONMENT INTERFACE AMONG FARMING COMMUNITIES IN WAKISO DISTRICT, UGANDA"

All the other conditions and responsibilities identified on your **Provisional Admission** Letter of **26th July, 2021`** such as annual renewal of registration, supervisors, fees and registration process remain unchanged.

I congratulate you on gaining **Full Admission** to Makerere University to study for a Ph.D. degree and on behalf of the University; I extend to you a warm welcome and wish you success in your studies.

Yours faithfully,

Buyinza Mukadasi
<u>PROFESSOR and DIRECTOR</u>
MB/Mm

cc: The Academic Registrar, Makerere University
" The Principal, College of **<u>HEALTH SCIENCES</u>**
" The Dean, School of **<u>PUBLIC HEALTH</u>**
