# Supplementary material for: Epidemiology of extended-spectrum beta-lactamase-producing Escherichia coli at the human-animal-environment interface in a farming community of central Uganda
Source: PLOS Glob Public Health. 2023 Jun 13;3(6):e0001344. doi: 10.1371/journal.pgph.0001344 (PMC10270331; doi:10.1371/journal.pgph.0001344)
Supplement: Attachment — Submitted filename: Response to reviewers.pdf [file pgph.0001344.s004.pdf]

Journal Requirements:

Response to comments in manuscript (PGPH-D-22-01776R1)

1. Please amend your detailed Financial Disclosure statement. This is published with the article. It must therefore be completed in full sentences and contain the exact wording you wish to be published.

a. State the initials, alongside each funding source, of each author to receive each grant.

Authors: The corresponding author **(JM)** received funding from CARTA and CIDIMOH to execute the study.

b. State what role the funders took in the study. If the funders had no role in your study, please state: “The funders had no role in study design, data collection and analysis, decision to publish, or preparation of the manuscript.”

Authors: “The funders had no role in study design, data collection and analysis, decision to publish, or preparation of the manuscript.”

c. If any authors received a salary from any of your funders, please state which authors and which funders.

Authors: No authors received a salary

If you did not receive any funding for this study, please simply state: “The authors received no specific funding for this work.”

Authors: A clear funding statement has been added.

2. Please upload all main figures as separate Figure files in .tif or .eps format only. For more information about how to convert and format your figure files please see our guidelines:

https://journals.plos.org/globalpublichealth/s/figures

Authors: All figures have been revised accordingly into the .tif

3. In the online submission form, you indicated that "Data will be made available upon request". All PLOS journals now require all data underlying the findings described in their manuscript to be freely available to other researchers, either 1. In a public repository, 2. Within the manuscript itself, or 3. Uploaded as supplementary information. This policy applies to all data except where public deposition would breach compliance with the protocol approved by your research ethics board. If your data cannot be made publicly available for ethical or legal reasons (e.g., public availability would compromise patient privacy), please explain your reasons by return email and your exemption request will be escalated to the editor for approval. Your exemption request will be handled independently and will not hold up the peer review process, but will need to be resolved should your manuscript be accepted for publication. One of the Editorial team will then be in touch if there are any issues.

Authors: A dataset has been added as supplementary 1 file.

Authors: All journal requirements have been put into consideration.

Reviewer #1: 1-Recent references are recommended.

Authors: A clear update has been made to the list of references

2-Why authors did not use PCR as confirmatory tool for detection of ESBL genes???, phenotypic detection of ESBL strains is not enough.

Authors: PCR is not a confirmatory tool as it also serves as a screening tool which is slightly more expensive. Different ESBL organisms produce several genes each of which requires unique primers. We used a modified double disc diffusion which has a good sensitivity and specificity based on earlier studies in the identification of ESBL producers. We also work from a low and middle income country with low resource settings. We aim at guiding public health as well as human and veterinary practice using affordable microbiological technologies which can be utilised even after our study. We however recommend advanced molecular tools for future studies to characterize the strains and types of ESBL genes present to guide on prevention and control of transmission.

3-Separation between conclusion and recommendations is necessary.

Authors: We have separated these two sections. However, based on previously published articles in the journal, many have not separated the two.

4-Clear strategies in order to protect human, animal and environment from ESBL are needed.

Authors: These have been added in the recommendation section of the revised manuscript.

Reviewer #2: The significance of the One Health concept in the fight against AMR cannot be overstated, thus the significance of this study. It is suggested that authors review the following as stated in the comments:

Line 124- What quality standards were utilized to eliminate obtained samples?

Authors: Some samples i.e. urine, water, faecal containers were not tied well so they had uncapped. So any batch that had one of the sample containers opened or sample spilled was

rejected. Secondly, any samples that never had accompanying data i.e. sample location, characteristics, household number, among others led to a rejection of the entire batch since such samples could not be easily matched with the household data collected using questionnaires to assess the epidemiological factors (Lines 266 - 272).

After obtaining pure cultures, samples, used culture media and other apparatus were decontaminated by autoclaving at 121°C for 60 minutes. Then reusable cleaned for recycling and non-reusable including samples and media disposed of safely by incineration.

Line 129- Address the multistage sampling strategy referred to as "figure 1" on line 129 is nowhere further included in the text. While "figure 1" on line 170 depicts primary and sub-cultures.

Authors: This was an oversight. Thank you, the word figure 1 has been removed as a detailed description of the sampling strategy in lines 131 – 142.

The entire Table and figure labelling should be addressed and corrected. (Lines 264, 265 & 296)

Authors: Table 3 and figure have been revised accordingly.

Line 149 & 205- The process of collecting samples needs additional elaboration and explanation. There was a discrepancy between the statement in line 205 that research assistants are responsible for sample collection after receiving proper training and the instruction provided to study participants regarding collection procedures in line 149.These require clarification for proper comprehension.

Authors: Clarity has been added in line 210- 213 on who collected which samples.

Line 160-How were the samples stored before they were processed in the laboratory, and was the processing done as soon as the samples arrived in the laboratory?

Authors: We had two teams i.e. the field teams and the laboratory teams. Samples were processed immediately as soon as they arrive in the laboratory within 4 hours of collection. We ensured this by collecting fewer samples at every collection day that can be easily

processed on the same day. Transport media was used to collect swab samples and cold chain was maintained by use of cool boxes. The original samples after culture were then kept in a fridge at 4°C for reference in case any of the cultures was not successfully done. (Lines 166 – 171)

Line 242- Were some practice questions administered, observed, or reported by the participants? If they were reported, those should be stated and possibly signified as a limitation of the study since participants were not observed carrying out those practices.

Authors: We assessed practice through questionnaires hence reported than observed. This is because some of these practices happen at specific times of the day and for a given reason. Our presence could even bias the way the practice is done. Therefore we created good rapport which yielded results as close as those that would be observed. Therefore, statements on practice assessment has been added as a limitation of the study in lines 427-431.

Lines 241 - 242 & 259- Error referencing in results should be clarified.

Authors: This has been revised accordingly

Lines 271- 273- The sentence should be restructured for ease of understanding and clarity.

Authors: This has been revised accordingly. Thank you

Line 296- All tables should be re-formatted, and authors should consider reporting only adjusted PR instead of having unadjusted PR in the table.

Authors: This has been looked into. However the authors felt like there was need to guide our readers on how the progress of model development went through bivariate analysis to multivariate. This would provide good guidance for all readers of the manuscript after it is published. Thank you so much for the guidance.

To correct all grammatical and spelling errors, the entire manuscript should be reviewed by a native English speaker.

Authors: A thorough English check has been done on the document.

Reviewer #3: Muleme et al., studied the Epidemiology of Extended-spectrum beta-lactamase-producing Escherichia coli at the human-animal-environment interface in Wakiso district, Uganda.

My comments are as follows:

Abstract

1. Line 38. Replace the word explained and write in past tense. Eg examined

Authors: This has been revised accordingly. Thank you

2. Line 48. You cannot have a total of percentage. Rephrase as a total of ** households or write the percentage and remove the "a total of"

Authors: This has been revised accordingly. Thank you

3. Line 53. You never mentioned the word "gardening" in the entire manuscript. Kindly check line 387.

Authors: This has been revised accordingly. Thank you

Introduction

1. Reference 1 is on Uganda NPHC report. I DO NOT think that report is the correct reference for line 67-69.

2. Line 71. Reference 2 is missing

3. Line 72. Rephrase this statement as: Studies have reported that the resistance to third generation cephalosporins in E. coli ranged from 0–87% whereas resistance to fluoro……..

Authors: This has been revised accordingly. Thank you

4. Line 74. Change and colleagues to "et al"

Authors: This has been revised accordingly. Thank you

5. Co-carriage with what? Fluoroquinolones?

Authors: This meant co-carriage of ESBL PE among individuals in the same household

6. Materials and Methods

Materials and Methods

7. Line 103. Rephrase as: our study "described" and remove "was formulated aiming at".

Authors: This has been revised accordingly. Thank you

8. Line 110. Include "average" before household and include unit such as 4.7 persons.

Authors: This has been revised accordingly. Thank you

9. Line 118-127. Could you justify why 260 samples (1248-988) were sub-optimal and did not pass your "thorough quality check"?

Authors: Some samples i.e. urine, water, faecal containers were not tied well so they had uncapped. So any batch that had one of the sample containers opened or sample spilled was rejected. Secondly, any samples that never had accompanying data i.e. sample location, characteristics, household number, among others led to a rejection of the entire batch since such samples could not be easily matched with the household data collected using questionnaires to assess the epidemiological factors. Lines (139 – 143) describe this process.

10. If your formulae gave you 840 samples, why did you over-sample and collected 1248 out of which 260 were bad and you ended up with 988?

Authors: We considered sample size to mean the minimum number of samples to be picked. Increasing the sample size raised the power of our study. Rejection of 260 samples was done following a quality control process. All the 988 samples retained had passed the quality checks, had all the required data and were processed and results reported in this manuscript.

11. Line 129. Change secondary samples to secondary sampling units.

Authors: This has been revised accordingly. Thank you

12. Line 143. Is the word recruited appropriate for households?

Authors: This has been replaced with "studied"

13. Kindly state that no personal identifying information such as name, phone number

or other materials were collected. If they were collected, how did you ensure their protection?

Authors: We have added information on this in lines (247 – 251)

14. Line162. Include the country of the Chromoger.

Authors: The country (Spain) was added.

15. Line 182. Most labs would culture MHA meant for AST at 35 degree and not 37. Kindly confirm this.

Authors: This has been revised accordingly. Thank you

Results

16. Line 226. Add “and” before 71.1%

Authors: This has been revised accordingly. Thank you

17. Table 1. Factor number 11 and 13 do not add up to 104. Cross-check these.

Authors: This has been revised accordingly. Thank you

18. Line 232. How did you arrive at 196 participants? One would assume that 4 individual per household (n=104) would result in 416 human participants “(samples).

Authors: In each household, we targeted to sample 2 individuals. Each of these individuals was to provide a urine and a faecal sample. During sampling, some people could not obtain a faecal sample and only provided urine and vice versa. During collection and transportation, some samples were lost and others retained after quality checks. The expected number would be (104X2 i.e. 2 people) which makes it 208 humans however, we only managed to obtain 196 participants. This implies, for the 12 participants missing, some could be due to the fact that their samples were rejected or perhaps only one individual in a household provided a sample instead of two people.

19. Line 232. Please remove were recruited to provide samples.

Authors: This has been revised accordingly. Thank you

20. Line 236. It would be difficult to separate stomach from intestinal. SO, I would suggest gastrointestinal illnesses rather than intestinal.

Authors: This has been revised accordingly. Thank you

21. Line 236. Change "had been commonly suffering" to "have previously experienced"

Authors: This has been revised accordingly. Thank you

22. Line 238-239. Kindly include the % in the bracket before the fraction.

Authors: This has been revised accordingly. Thank you

23. Line 241-242. Correct the error in the Table.

Authors: This has been revised accordingly. Thank you

24. Line 243. Shoat does not mean sheep and goat. Kindly confirm the meeting and edit appropriately.

Authors: This has been revised accordingly. Thank you

25. Line 253. Change "up to" to "approximately"

Authors: This has been revised accordingly. Thank you

26. Line 259. Revise the highlighted "Error"

Authors: This has been revised accordingly. Thank you

27. Line 264. Include Figure number

Authors: This has been revised accordingly. Thank you

28. Line 265. Delete Figure from title of table, Delete and households too in Title.

Authors: This has been revised accordingly. Thank you

29. Line 277-278 and Line 286-287 appears as a repetition to me. Could you please differentiate these statements?

Authors: This has been revised and clarity made on the difference between the two sentences. Line 277-278 refers to human carriage while Line 286-287 refers to household contamination. These have been corrected

30. Line 290. Kindly report in past tense. Those that "used" not "using"

Authors: This has been revised accordingly. Thank you

31. Line 296. Correct table title.

Authors: This has been revised accordingly. Thank you

32. Line 296. Table seems unconvincing. A. Could you tell me what your outcome variable was? B. Was the LR model based on presence of ESBL-PE or absence of ESBL-PE?

Authors: There were two outcome variables i.e. human carriage and household contamination. Human carriage meant that each household that had a positive human sample i.e. either urine or faecal or both was considered positive and the opposite was true for other households without positive human samples. Household contamination meant those households that had at least one ESBL-PE positive isolate. The LR model was based on presence of ESBL-PE

C. Could you tell me why a household with dirty environment could have less prevalence than a clean household or why drugs administered by a Vet could have resulted in more ESBL than if it were administered by non-vets?

Authors: Households with dirty environments; the argument here was that dirt in the eyes of a researcher or inspector does not necessarily imply bacterial contamination. This helps us when designing public health interventions to know that even if a surface, water or anything like food visually looks clean, it does not guarantee safety. Those that look dirty also do not necessarily translate into contamination. Working with veterinarians was found associated with high ESBL-PE contamination and carriage because these Vet do not observe IPC and Biosecurity measure between farms. He can use the same re-usable syringe on animals in one farm and on animals on another firm without disinfecting. They can spend a whole day moving from one farmer to another treating animals and or vaccinating without a proper disinfectant. Therefore sensitization and increased biosecurity on farm would be ideal for changing such a situation. Our farmers actually have a big trust in their vets, therefore will

pay less attention to biosecurity even when a vet is suspected to be contaminated

DISCUSSION

33. Line 316. Change round to around

Authors: This has been revised accordingly. Thank you

34. Line 322. Change as well as to "and could compromise…"

Authors: This has been revised accordingly. Thank you

35. Line 325. Include "the" before the least contributor.

Authors: This has been revised accordingly. Thank you

36. Rephrase line 357. I suggest: "This is similar to the report of Brick et al who reported that …….

Authors: This has been revised accordingly. Thank you

37. Your references are not formatted to the Vancouver Standard.

Authors: This has been revised to Vancouver standard. Thank you 38. Your last reference was 30. However, from Line 335, you have continued with 31, 32 etc. So, update your reference list and format correctly.

Authors: This has been revised and updated. Thank you

---

6. PLOS authors have the option to publish the peer review history of their article (what does this mean?). If published, this will include your full peer review and any attached files.

Authors: Yes

Do you want your identity to be public for this peer review? If you choose "no", your identity will remain anonymous but your review may still be made public.

Authors: Yes
