# Supplementary material for: Epidemiology of extended-spectrum beta-lactamase-producing Escherichia coli at the human-animal-environment interface in a farming community of central Uganda
Source: PLOS Glob Public Health. 2023 Jun 13;3(6):e0001344. doi: 10.1371/journal.pgph.0001344 (PMC10270331; doi:10.1371/journal.pgph.0001344)
Supplement: Attachment — Submitted filename: Response to reviewers.docx [file pgph.0001344.s005.docx]

# SCHOOL OF PUBLIC HEALTH

**Department of Disease Control and Environmental Health**

29^th^ March 2023

Dear Editor

PLOS Global Public Health

RE: Epidemiology of Extended-spectrum beta-lactamase-producing Escherichia coli at the human-animal-environment interface in a farming community of central Uganda

I hereby submit our revisions to PLOS Global Public Health for our manuscript “PGPH-D-22-01776R1” for your consideration. We have made thorough changes and also made a critical change of the acronym of Extended Spectrum Beta-lactamase producing Escherichia coli from ESBL-PE to ESBL-Ec after checks with different earlier authors and the World Health Organization documents.

Looking forward to publishing with this prestigious journal. We feel we have extensively addressed all the reviewer comments. Thank you


Kind regards,

Muleme James (On-behalf of the co-authors)

Makerere University School of Public Health

+256787364697

**Comments and responses**

Journal Requirements:

Please review your reference list to ensure that it is complete and correct. If you have cited papers that have been retracted, please include the rationale for doing so in the manuscript text, or remove these references and replace them with relevant current references. Any changes to the reference list should be mentioned in the rebuttal letter that accompanies your revised manuscript. If you need to cite a retracted article, indicate the article’s retracted status in the References list and also include a citation and full reference for the retraction notice.
Additional Editor Comments (if provided):
[Note: HTML markup is below. Please do not edit.]
Reviewers' comments:
Reviewer's Responses to Questions

**Authors response:** Thank you. We have followed all the journal requirements for publication.

**Comments to the Author**
1. If the authors have adequately addressed your comments raised in a previous round of review and you feel that this manuscript is now acceptable for publication, you may indicate that here to bypass the “Comments to the Author” section, enter your conflict of interest statement in the “Confidential to Editor” section, and submit your "Accept" recommendation.

Reviewer #2: (No Response)

Reviewer #3: (No Response)

**Authors response:** Thank you

2. Does this manuscript meet PLOS Global Public Health’s [publication criteria](https://journals.plos.org/globalpublichealth/s/criteria-for-publication)? Is the manuscript technically sound, and do the data support the conclusions? The manuscript must describe methodologically and ethically rigorous research with conclusions that are appropriately drawn based on the data presented.

Reviewer #2: Yes

Reviewer #3: Partly

**Authors response:** Thank you

3. Has the statistical analysis been performed appropriately and rigorously?

Reviewer #2: Yes

Reviewer #3: Yes

**Authors response:** Thank you

4. Have the authors made all data underlying the findings in their manuscript fully available (please refer to the Data Availability Statement at the start of the manuscript PDF file)?

The [PLOS Data policy](https://journals.plos.org/globalpublichealth/s/data-availability) requires authors to make all data underlying the findings described in their manuscript fully available without restriction, with rare exception. The data should be provided as part of the manuscript or its supporting information, or deposited to a public repository. For example, in addition to summary statistics, the data points behind means, medians and variance measures should be available. If there are restrictions on publicly sharing data—e.g. participant privacy or use of data from a third party—those must be specified.

Reviewer #2: Yes

Reviewer #3: Yes

**Authors response:** Thank you

5. Is the manuscript presented in an intelligible fashion and written in standard English?
PLOS Global Public Health does not copyedit accepted manuscripts, so the language in submitted articles must be clear, correct, and unambiguous. Any typographical or grammatical errors should be corrected at revision, so please note any specific errors here.

Reviewer #2: Yes

Reviewer #3: Yes

**Authors response:** Thank you

6. Review Comments to the Author

Please use the space provided to explain your answers to the questions above. You may also include additional comments for the author, including concerns about dual publication, research ethics, or publication ethics. (Please upload your review as an attachment if it exceeds 20,000 characters)

**Reviewer #2: We appreciate your efforts to correct all observed comments. However, kindly address the following observations as well.**

Line 125- How was cross-contamination avoided if samples from humans, the environment, and animals were kept in the same plastic bag?

**Authors response:** Original samples were placed in small Ziploc bags (i.e., each sample type was placed in a unique Ziploc bag). After we placed these already safely packed Ziploc bags into one plastic bag big enough to contain the entire batch to represent a household. For any leakage, it was evident that a particular Ziploc bag i.e. may be for human feces, urine, or environmental sample or even animal fecal sample Ziploc bag had spilled. Therefore, we discarded such.

Line 154- Explain the process of selecting one individual in a selected household who was requested to provide a fecal and urine sample.

**Authors response:** We have corrected that it was a minimum of one individual and a maximum of two individuals within a household that provided fecal and or urine samples at their convenience. We targeted individuals who spent much time home, taking care of domestic animals in that particular household. For children under 18 years, after seeking consent and assent, we chose those that played mostly with animals at home and environmental areas that were near animal houses at the time of sampling.

Results section- Tables need to be adequately labelled.

**Authors response:** Thank you. We have made some changes to the labels for the tables in the results section.

**Reviewer #3: You have diligently reviewed the manuscript. Congratulations.
Authors response:** Thank you.

Could you look into the following.
Line 51. As much as possible, dont use the word ”However”. So, delete and start the statement with ””Covering the ....”.

**Authors response:** Thank you. This has been addressed as per your guidance
Line 70. A review by ……… cannot have three references. I suggest you change to studies conducted in “xxx country” showed

**Authors response:** Thank you. We have removed references 7 and 8 and retained one that the statement refers to.
Line 75. Relationships or interactions?

**Authors response:** Thank you. This has been changed to interactions
Line 78 & 79. Italicize E.coli

**Authors response:** Thank you. This has been done accordingly

Line 82. Change since different studies to ….. as different studies have recognized the role of the environment ….. Kindly write in past tense as much as possible

**Authors response:** Thank you. This has been revised to past tense as guided
Line 117. Am I right to assume that you wanted to sample 104 households, you got 12 samples from each (total of 1248 and 988 passed your sample screening test). Where does the 840 come from?
**Authors response:** Thank you. Originally, we had calculated a sample size of 840 samples however, that was counted as the minimum number of samples to be picked for our study. This implied that beyond these samples, our study would benefit from a high power. In this correction, we have removed the 840 samples (hypothetically calculated) and retained the 1248 and 988 which were actually done.

Line 118. Delete the “,” after (23)

**Authors response:** Thank you. The entire sentence where this was has been removed from the revised manuscript.

Line 132. Change approach to technique

**Authors response:** Thank you. This has been addressed accordingly

Line 149. Rephrase as : mobile based application (kobo Toolbox).

**Authors response:** Thank you. This has been addressed accordingly

Line 176. The wrong word: incubated. Do you mean : freshly prepared?

**Authors response:** Thank you. This has been revised accordingly

Line 180, 193. Use o instead as the 0 is wrong

**Authors response:** Thank you. This has been addressed accordingly

Line 185. Standard protocols or manufacturer instructions and not standard operating procedures.

**Authors response:** Thank you. This has been revised accordingly and we have adopted the phrase manufacturer’s instructions

Line 242. Remove the bracket on “5-9 members”

**Authors response:** Thank you. This has been removed accordingly

Line 154 said : one individual was requested to provide samples per household, Now line 249. You said 196 study participants, How did this come about? Kindly reconcile

**Authors response:** Thank you. We have corrected that sample size issue in line 154 to “a minimum of 1 and maximum of 2 individuals in a household were requested to provide samples”. This implies that the expected number would be 208 human participants to provide samples. We however ended up with 196 human participants getting a deficit of 12 participants. These either rejected their participation, we never got a second person in the sampled household or their samples were contaminated and therefore could not be used in the analysis.

Line 257-258, 280, 287 – Typographical error in Table title

**Authors response:** Thank you. Thorough revisions have been made on table titles.

Line 255 – You said shoats – 99/393 and in Table pig is 78… you need to reconcile this
Table ****…. ANIMAL SUBJECTS: I think the denominator should be the number of household and not number of animals. Do you think there would be difference in husbandry practice, feeding, source of water, and treatment option per animal belonging to the same household. I think it will make more sense if its based on household and not animals. Instead of 321 animals being treated by a Vet, you state **% of animal owners consulted a vet to treat their animals. What do you think?

**Authors response:** Thank you. We had forgotten to put a table legend for shoats. We have however revised this to capture this well as these meant a combination of sheep and goats which normally are reared together under similar conditions and husbandry practices. It is important to note, that within a household, animals are given different levels of attention and prioritization given their role in that household, i.e. animals that bring in money are at many times given better facilities and attention when compared to other animals. Therefore, the attachment of value to an animal within a household makes differences in the variables assessed. Our intention for this table was to get the dynamics and descriptive statistics for the study subjects i.e. humans versus animals but not intentions for relations since they are catered for under multi-variable analysis and also not our target outcomes. We however agree that for a study that was designed in the initials stages to compare and look at the dynamics of animals in relation to their owners would benefit more. Our main aim was on prevalence, severity of ESBL-Ec as well as the factors associated to this outcome including the distribution.

Line 260-266. Is repetition of methods and no results therein. Consider deleting it or move to methods. Check Line 123 and 159-160.
**Authors response:** Thank you. We have agreed to delete the section from the manuscript as it does not fit well in the methods section.

Line 269. This was result of analysis of field samples. Why do you need a CI:. You don’t need CI for isolates retrieved from samples.

**Authors response:** Thank you for the information and advise. This has been addressed accordingly

Line 291. AN opening statement would make it easier to read. Something like: several factors (recent visitors, source of water, use of protective lid for water, etc) were associated with ESBL-PE human carriage among the study population.

**Authors response:** Thank you. This statement has been added as an opening phrase to the section.

Line 291-293 Contradicts line 299-301. Why the results (OR and 95% CI) different?

**Authors response:** Thank you for the information and advise. In table 4, we present results from two outcome variables i.e. human carriage of ESBL-Ec and Household contamination with ESBL-Ec. During analysis, we found the same factors being associated to both outcomes even though the strength of this associated varied. This brings the discrepancy in the results as the reviewer has stated in their comment.

Line 317. Please remove “the” before LMICs
**Authors response:** Thank you for the advice. This has been addressed accordingly.

Line 320. Rephrase the statement to begin as: “Our findings showed that two factors: visit by animal health workers and visitation to a human health facility were highly associated……. “

**Authors response:** Thank you for the advice. This has been addressed accordingly.

Line 353-356. Looks like repetition despite the differences: sample collection and data collection. That then raises the question why did you collect them on different days? I suggest you delete one to save you some stress.

**Authors response:** Thank you for the comment. We present results of two outcomes i.e. household contamination and human carriage for ESBL-Ec. We have re-arranged the sentences in the lines above to start with households and then humans within such households that had a visitor in the previous 24 hours before sample collection. We have maintained sample collection as this were done together with data collection. So we have deleted data collection in that regard. This has been addressed accordingly.

Line 362. Am i correct that COVID19 or COVID-19 is the most common way of writing

**Authors response:** Thank you for the advice. This has been addressed accordingly.

Line 375. Since you mentioned the study... you should state the different views and contentions as argued by that study.

**Authors response:** We have a sentence that reads “Several studies have indicated that good hygienic practices, increased household use of PPE and low abundance of bacterial pathogens in the environment could potentially lead to reduced contamination especially with ESBL-PE organisms (31,40).” In there, we try to present the different factors (views or contentions) argued by the different writers. Therefore we agree with the need to do so as suggested in the comment. Thank you.

Line 387. Kindly rephrase as: ... were at higher risk of contracting ESBL-PE and other nosocomial infections (41).

**Authors response:** Thank you for the advice. This has been addressed accordingly.

Am happy to recommend the manuscript if these observations have been addressed.
**Authors response:** Thank you

**Comments from the portal**

1. Recent references are recommended

**Authors response:** Thank you. We have revised the list of references accordingly. The only old reference is for an article that is relevant and no other has been published in the same regard with similar content.

2. Why authors did not use PCR as confirmatory tool for detection of ESBL genes???, phenotypic detection of ESBL strains is not enough

**Authors response:** We conducted this study in a low- and middle-income country with limited resources, i.e., there are only a few health care facilities with capacity to conduct cultures and microbial identification. Molecular methods are only in a few laboratories especially research laboratories which rarely contribute to supporting treatment in healthcare facilities. We appreciate the need to conduct further molecular and or genotypic level analysis. However, this was beyond the scope of the study. Molecular methods like phenotypic methods also have several shortcomings. However, the use of a given method depends on the;

a) Settings: For the case of Uganda, the country does not have the capacity to conduct molecular methods at a large scale, because, they are expensive, and the technology is not readily available in most laboratories. It only exists in certain laboratories and come with a high cost. This implies that healthcare facilities can mainly access phenotypic or culture methods and the cheap Rapid diagnostic tools for most diagnostics. Use of molecular methods may not be helpful to support the healthcare system of a country like Uganda since these are not present in most facilities.
b) Research question are set to guide a given research process. However, research questions in a given setting will also be set to be answered by the available methods.
3. Separation between conclusion and recommendations is necessary

**Authors response:** Thank you. This has been addressed accordingly

4. Clear strategies in order to protect human, animal and environment from ESBL are needed

**Authors response:** Thank you. This has been addressed accordingly
