# Supplementary material for: Epidemiology of extended-spectrum beta-lactamase-producing Escherichia coli at the human-animal-environment interface in a farming community of central Uganda
Source: PLOS Glob Public Health. 2023 Jun 13;3(6):e0001344. doi: 10.1371/journal.pgph.0001344 (PMC10270331; doi:10.1371/journal.pgph.0001344)
Supplement: Attachment — Submitted filename: Response to reviewers comments.docx [file pgph.0001344.s006.docx]

Reviewer's Responses to Questions

**Comments to the Author**

1. If the authors have adequately addressed your comments raised in a previous round of review and you feel that this manuscript is now acceptable for publication, you may indicate that here to bypass the “Comments to the Author” section, enter your conflict of interest statement in the “Confidential to Editor” section, and submit your "Accept" recommendation.

Reviewer #2: (No Response)

Reviewer #3: All comments have been addressed

Reviewer #4: All comments have been addressed

**Authors**: Thank you

2. Does this manuscript meet PLOS Global Public Health’s [publication criteria](https://journals.plos.org/globalpublichealth/s/criteria-for-publication)? Is the manuscript technically sound, and do the data support the conclusions? The manuscript must describe methodologically and ethically rigorous research with conclusions that are appropriately drawn based on the data presented.

Reviewer #2: Partly

Reviewer #3: Yes

Reviewer #4: Yes

**Authors**: Thank you

3. Has the statistical analysis been performed appropriately and rigorously?

Reviewer #2: Yes

Reviewer #3: Yes

Reviewer #4: Yes

**Authors**: Thank you

4. Have the authors made all data underlying the findings in their manuscript fully available (please refer to the Data Availability Statement at the start of the manuscript PDF file)?

The [PLOS Data policy](https://journals.plos.org/globalpublichealth/s/data-availability) requires authors to make all data underlying the findings described in their manuscript fully available without restriction, with rare exception. The data should be provided as part of the manuscript or its supporting information or deposited to a public repository. For example, in addition to summary statistics, the data points behind means, medians and variance measures should be available. If there are restrictions on publicly sharing data—e.g., participant privacy or use of data from a third party—those must be specified.

Reviewer #2: Yes

Reviewer #3: Yes

Reviewer #4: Yes

**Authors**: Thank you

5. Is the manuscript presented in an intelligible fashion and written in standard English?

PLOS Global Public Health does not copyedit accepted manuscripts, so the language in submitted articles must be clear, correct, and unambiguous. Any typographical or grammatical errors should be corrected at revision, so please note any specific errors here.

Reviewer #2: Yes

Reviewer #3: Yes

Reviewer #4: Yes

**Authors**: Thank you

6. Review Comments to the Author

Please use the space provided to explain your answers to the questions above. You may also include additional comments for the author, including concerns about dual publication, research ethics, or publication ethics. (Please upload your review as an attachment if it exceeds 20,000 characters)

Reviewer #2: Thank you for the great improvement and work done thus far.

However, kindly pay attention and modify these few observations.
"Line 107" - Provide a reference to the statement made about Wakiso district.

**Authors:** Thank you, I reference has been added in line 108 of the revised version

"Line 132" - Reference this ((www.random.org?)) appropriately according to the publication criteria.

**Authors:** Thank you, the reference has been removed and instead a hyper-link inserted to the text. Otherwise, we never intended it to be a citation by itself.

"Line 137" - Clarify from which study participants (humans or animals) were you checking for the history of diarrhea episodes and specify how you ascertained the information.

**Authors:** Thank you. This was an oversight. We meant diarrhea from humans as reported by local leaders (“community gatekeepers")

"Line 225, 271, 280, 303" - Clarify the error response feedback (Error! Reference source not found.). throughout the paper.

**Authors:** We have made a thorough check in the entire manuscript, made revisions to the errors and we feel all the references are now okay.

"Line 272, 304" - Clarify the table error response (Table Error! Reference source not found), found throughout the manuscript.

**Authors:** Thank you. These have been corrected accordingly in the entire manuscript

Reviewer #3: 1, Line 298 and 301 still looks like repetition. It might be scientifically correct but same aOR looks odd.
**Authors:** True, they show a similar APR even though at bivariate they showed different unadjusted PRs. It is perhaps something to pick interest in future research. Could be because they are both professional or recommended sources of health service support for humans and animals may be they have same chances of impacting on the outcome of interest using the same sample.

2. Except for table 1, All table titles (and the line before it) have not be correctly written. –Contains the word *Error* - probably the author doesn’t see this, and we only see it during conversion to pdf

**Authors:** True, it was not visible in the word document, but the pdf could show. We have corrected all these errors.

Reviewer #4: Please edit every aspect of the manuscript where the words "source file not found" are appearing.

**Authors:** Thank you. These have been corrected accordingly in the entire manuscript

Though the authors have addressed the majority of the previous comments raised, I still feel strongly that there is a need for the limitation associated with this study be presented just before the conclusion. For instance, the authors did not characterize the ESBL isolates using molecular tools that would allow for adequate discrimination of the strains. This limitation, among others should be discussed.

**Authors:** Thank you. This has been added at the end of the discussion section where other limitations were discussed.

7. PLOS authors have the option to publish the peer review history of their article ([what does this mean?](https://journals.plos.org/globalpublichealth/s/editorial-and-peer-review-process#loc-peer-review-history)). If published, this will include your full peer review and any attached files.


**Do you want your identity to be public for this peer review?** If you choose “no”, your identity will remain anonymous but your review may still be made public.

**Authors:** Yes

For information about this choice, including consent withdrawal, please see our [Privacy Policy](https://www.plos.org/privacy-policy).

Reviewer #2: No

Reviewer #3: No

Reviewer #4: No

**Authors:** Thank you
